# BET protein inhibitor JQ1 inhibits growth and modulates WNT signaling in mesenchymal stem cells

**DOI:** 10.1186/s13287-016-0278-3

**Published:** 2016-02-01

**Authors:** Saeed Alghamdi, Irfan Khan, Naimisha Beeravolu, Christina McKee, Bryan Thibodeau, George Wilson, G. Rasul Chaudhry

**Affiliations:** Department of Biological Sciences, Oakland University, Rochester, MI 48309 USA; OU-WB Institute for Stem Cell and Regenerative Medicine, Oakland University, Rochester, MI 48309 USA; Beaumont Health System, Royal Oak, MI 48073 USA

**Keywords:** Thienodiazepine, JQ1, Mesenchymal stem cell, Growth inhibition, Cell cycle arrest, Gene regulation, WNT

## Abstract

**Background:**

Efficacy and safety of anticancer drugs are traditionally studied using cancer cell lines and animal models. The thienodiazepine class of BET inhibitors, such as JQ1, has been extensively studied for the potential treatment of hematological malignancies and several small molecules belonging to this class are currently under clinical investigation. While these compounds are well known to inhibit cancer cell growth and cause apoptosis, their effects on stem cells, particularly mesenchymal stem cells (MSCs), which are important for regeneration of damaged cells and tissues, are unknown. In this study we employed umbilical cord derived MSCs as a model system to evaluate the safety of JQ1.

**Methods:**

Cord derived MSCs were treated with various doses of JQ1 and subjected to cell metabolic activity, apoptosis, and cell cycle analyses using MTT assay, Annexin-V/FITC and PI staining, and flow cytometry, respectively. The effect of JQ1 on gene expression was determined using microarray and quantitative real-time reverse transcriptase polymerase chain reaction analysis. Furthermore, protein expression of apoptotic and neuronal markers was carried out using western blot and immunostaining, respectively.

**Results:**

Our results showed that JQ1 inhibited cell growth and caused cell cycle arrest in G1 phase but did not induce apoptosis or senescence. JQ1 also down-regulated genes involved in self-renewal, cell cycle, DNA replication, and mitosis, which may have negative implications on the regenerative potential of MSCs. In addition, JQ1 interfered with signaling pathways by down regulating the expression of WNT, resulting in limiting the self-renewal. These results suggest that anticancer agents belonging to the thienodiazepine class of BET inhibitors should be carefully evaluated before their use in cancer therapy.

**Conclusions:**

This study revealed for the first time that JQ1 adversely affected MSCs, which are important for repair and regeneration. JQ1 specifically modulated signal transduction and inhibited growth as well as self-renewal. These findings suggest that perinatal MSCs could be used to supplement animal models for investigating the safety of anticancer agents and other drugs.

**Electronic supplementary material:**

The online version of this article (doi:10.1186/s13287-016-0278-3) contains supplementary material, which is available to authorized users.

## Background

Recently, bromo- and extra-terminal domain (BET) protein inhibition has emerged as a therapeutic target for cancer, metabolic disorders, and inflammatory diseases [1–3]. BET proteins contain two related bromodomain (BD) motifs, BD1 and BD2, located at the amino acid terminal region, and one extra-terminal (ET) domain located either at the carboxyl terminal region of the short isoform and protein members or at the central region of the long isoform and protein members. The two BDs facilitate binding to the BET protein acetylated lysine residues on the histone tail, while the ET domain recruits transcription and epigenetic regulatory factors [3–7]. In mammals, there are four members belonging to the BET protein subfamily: BRD2, BRD3, BRD4, and BRDT. BET proteins have been shown to play vital roles in the pathogenesis of specific leukemia and viral associated cancers such as Kaposi sarcoma and cervical cancers [1, 3, 5, 7]. BRD2 has been shown to play a role in rheumatoid arthritis while BRD4 is implicated in immune diseases through regulation of nuclear factor-κB-dependent genes in response to an activated innate immune system and regulation of macrophage primary responsive genes [8]. Consequently, BET proteins have been targeted for controlling cancerous cell growth.

Several small molecules belonging to the thienodiazepine group that inhibit BET proteins [1–3, 5, 7, 8] have been developed. Four such molecules (I-BET 762, OTX-015, TEN-010, and CPI-0610) have been approved as anticancer agents for clinical trials [9, 10]. More recently another such small molecule, JQ1 ((S)-tert-butyl 2-(4-(4-chlorophenyl)-2,3,9-trimethyl-6H-thieno[3,2-f][1,2,4]triazolo[4,3-a][1,4]diazepin-6-yl)acetate), has been shown to have high specificity against BET proteins. JQ1 functions by displacing the BET proteins from the acetylated lysine residues and inhibiting their activities. Chromosomal translocation of BRD3 and BRD4 has been shown to contribute to pathogenesis of the NUT (nuclear protein in testis) midline carcinoma (NMC) [2, 4, 6]. Treatment of NMC with JQ1 resulted in G1 cell cycle arrest, apoptosis, and differentiation [11]. Recently, the effects of JQ1 have been studied in various cancerous cell lines and animal models [12–15]. These studies showed that JQ1 inhibits BRD4 function, which leads to cell cycle arrest and promotion of apoptosis in multiple myeloma cells, leukemia and lymphoma cells, and neuroblastoma cells by downregulating expression of translocated *c-MYC* which is involved in their pathogenesis [12, 14, 15]. JQ1 has also been shown to decrease proliferation and induce apoptosis in NF1-associated malignant peripheral nerve sheath tumors [13]. Similar observations have been shown in DNMT3A (DNA methyltransferase 3A) mutated leukemia where JQ1 inhibits the action of BRD4 and induces caspase 3/7-mediated apoptosis [16]. Moreover, JQ1 has been shown to be an effective drug to treat STAT5 (Signal transducer and activator of transcription 5) associated leukemia and lymphoma through inhibition of BRD2 function [17].

Although JQ1 and other members of the thienodiazepine class of BET inhibitors are well investigated using cancerous cells, their effect on normal cells—particularly adult stem cells such as mesenchymal stem cells (MSCs)—has not been investigated to our knowledge. Cord-derived MSCs are more primitive and display greater self-renewal potential compared with MSCs derived from adult sources. Unlike MSCs from adult sources such as bone marrow MSCs, cord-derived MSCs can be expanded to provide sufficient amount of cells for experimentation. Therefore, we selected cord-derived MSCs as a model system to investigate the effects of JQ1. We hypothesized that JQ1 could affect cell growth and gene expression of normal stem cells such as MSCs differently to its known effects on cancer cells. In this study, we showed that JQ1 induced cell cycle arrest in the G1 phase of MSCs, but unlike cancer cells did not promote apoptosis. We found JQ1 also downregulated genes involved in self-renewal, mitosis, and DNA replication. We propose that human MSCs could be used in addition to animal models to investigate the safety of anticancer agents; because MSCs play a significant role in tissue repair and regeneration, findings from this investigation may be directly relevant to humans.

## Methods

### Culture of MSCs

Human umbilical cord samples were obtained from consented healthy donors through the Beaumont Hospital BioBank and isolation of MSCs was carried out at Oakland University (Rochester, MI, USA) under approved protocols (HIC# 2012-101 and IRB# 400244, respectively). Human umbilical cord-derived MSCs were isolated and characterized in our laboratory. Briefly, the region between the cord and placenta was dissected, minced into approximately 1–2 mm pieces, and cultured in 75 cm^2^ culture flasks using growth medium (GM) containing Dulbecco’s modified Eagle’s (DMEM) with 4500 mg/ml glucose and 2 mM l-glutamine (Invitrogen, Carlsbad, CA, USA), supplemented with 10 % fetal bovine serum (Aleken Biologicals, Nash, TX, USA), and antibiotic solution (0.1 % gentamicin, 0.2 % streptomycin, and 0.12 % penicillin) (Sigma Aldrich, St. Louis, MO, USA). The culture medium was changed every 3 days until cells grew to 70 % confluency from explants. Cells were dissociated using TrypLE Select (Invitrogen), passaged in the GM to passage 3, characterized using flow cytometry (BD Canto II, analyzed by FACS Canto II; BD Biosciences, San Jose, CA, USA) and differentiated to multilineage. Adherent cells displaying fibroblastoid morphology and positive for CD44, CD90, CD73, CD29, and CD105 and negative for CD34 and CD45 in accordance with the ISCT standards [18] were considered MSCs and used in this study.

### Treatment of MSCs with JQ1

MSCs (passages 8–10) were seeded onto 0.1 % gelatin-coated culture plates, and propagated in GM at 37 °C in an atmosphere of 5 % CO_2_ in a humidified incubator overnight_._ Cells were then treated with various concentrations ranging from 0 to 500 nM JQ1 (Brander’s Laboratory, Harvard Medical School, Boston, MA, USA). JQ1 was dissolved in dimethyl sulfoxide (DMSO; Thermo Fisher Scientific, Waltham, MA, USA).

### Cell viability and proliferation assays

MSCs (2.5 × 10^4^ cells/25 cm^2^ flasks) were grown overnight and treated with 0, 10, 100, and 500 nM JQ1 for 24 and 72 hours. Cells were dissociated with TrypLE, centrifuged, and resuspended in trypan blue solution for counting by hemocytometer. Cells stained blue were considered nonviable cells. The samples were analyzed in triplicate.

Cell proliferation and metabolic activity was determined by direct counting using the hemocytometer and MTT assay, respectively. For the MTT assay, cells were cultured in 24-well plates overnight and then incubated with 500 μg/ml MTT reagent (Sigma, St. Louis, MO, USA) at 37 °C for 2 hours to obtain blue formazan crystals. The formazan complex was solubilized by adding 150 μl isopropanol/HCl (15:1) to each well and the absorbance was determined at 570 nm using an Epoch Microplate Spectrophotometer (BioTek, Winooski, VT, USA).

### Immunocytochemistry assay

MSCs grown in GM containing 0 and 500 nM JQ1 for 5 days were rinsed with phosphate-buffered saline (PBS) and fixed with 4 % paraformaldehyde for 10 minutes at room temperature. Cells were washed with PBS, and then permeabilized with 0.5 % TritonX100 in PBS for 10 minutes. They were then washed twice with PBS followed by incubation with 2 % bovine serum albumin in PBS to block nonspecific binding for 30 minutes at room temperature. After blocking, cells were incubated in TUJ1 and MAP2 primary antibodies (diluted 1:50; Santa Cruz Biotechnology, Santa Cruz, CA, USA) overnight at 4 °C. Next, cells were washed three times with PBS for 5 minutes each time and were incubated in Cy3-labeled goat anti-mouse IgG and fluorescein isothiocyanate (FITC)-labeled goat anti-rabbit IgG (diluted 1:200; KPL, Gaithersburg, MD, USA), respectively, for 1 hour at room temperature, washed three times with PBS for 5 minutes each time, incubated with 4′,6-diamidino-2-phenylindole (DAPI; diluted 1:100) for nucleic acid staining for 10 minutes, and visualized by fluorescence microscopy (NIKON Instruments Inc., Melville, NY, USA).

### Apoptosis analysis

MSCs (1 × 10^6^ cells) were cultured in 75 cm^2^ flasks in GM overnight. Cells were exposed to 0, 10, 100, or 500 nM JQ1 for 24 and 72 hours. Cells were then harvested by dissociating with TrypLE, pelleted by centrifugation, washed with PBS, and resuspended in PBS. Cell suspensions were stained with Annexin V/FITC and propidium iodide (PI) according to the manufacturer’s instructions (Biolegend, San Diego, CA, USA). Briefly, PBS-washed cells were suspended in 100 μl FITC binding buffer at a minimum concentration of 1 × 10^6^ combined with 5 μl Annexin V/FITC and 10 μl PI (Sigma). After 15 minutes of incubation in the dark on ice, cells were centrifuged at 2000 rpm for 10 minutes, resuspended in PBS, and analyzed by FACSCanto II. Cells that were Annexin V-negative and PI-negative were considered viable cells. Cells positive for Annexin V only were considered apoptotic, and cells positive for PI only were considered necrotic or late apoptotic. All samples were prepared in triplicate.

### Cell cycle analysis

MSCs (1 × 10^6^ cells) were cultured in 75 cm^2^ flasks in GM overnight. Cells were exposed to DMSO and 0, 10, 100, or 500 nM JQ1 for 24 and 72 hours. Treated cells were harvested and fixed with 70 % ice-cold ethanol overnight at 4 °C. Control and treated cells were incubated with 1 mg/ml PI solution (Sigma), 0.5 % TritonX100, RNase, and PBS at room temperature in the dark for 20 minutes. Cells were then washed and resuspended in PBS. The DNA content was assessed using FACS Canto II and the results were analyzed using ModFit software (Verity Software House, Topsham, ME, USA). All samples were prepared in triplicate.

### Microarray analysis

MSCs (1 × 10^6^ cells) were cultured in 75 cm^2^ flasks in GM overnight. Cells were treated with 0, 100, and 500 nM JQ1 for 24 hours. RNA was isolated from frozen cell pellets using the E.Z.N.A. Total RNA Kit I (Omega, Norcross, GA, USA). RNA was purified using spin cartridge technology, quantified (Nanodrop 8000; Thermo Scientific), and stored at –80 °C. RNA was then amplified and labeled using the TargetAmp-Nano Labeling Kit (Epicenter, Madison, WI, USA) which enables amplification and target preparation compatible with the Direct Hybridization Assay (Illumina, San Diego, CA, USA). Amplification was performed with 500 ng total RNA input following procedures described in the TargetAmp-Nano Labeling Kit user guide. Hybridization and staining to the HumanHT-12 v4 Expression BeadChip (Illumina) was performed using 750 ng biotin-antisense RNA product following protocols outlined in the Whole-Genome Gene Expression Direct Hybridization Assay Guide (Illumina). Subsequent scanning of the BeadChip was performed using the iScan System (Illumina). Gene expression data were imported into Illumina Genome Studio (v2011.1) and subsequently analyzed in Partek Genomics Suite, Partek Inc. , St. Louis, MO, USA (6.6 version 6.14.0828). The accession number for the microarray data reported is [NCBI GEO:GSE705770].

### Quantitative reverse transcriptase PCR

Total cellular mRNA was isolated using the GeneJET RNA purification Kit (Thermo Fisher Scientific), following the manufacturer’s instructions. Total RNA was purified by incubation with DNase at 37 °C for 30 minutes using a thermocycler (MJ Research PTC-100 Thermal Cycler; GMI, Ramsey, MN, USA). cDNA was synthesized using the BioRad iScript kit (Bio-Rad, Hercules, CA, USA). Quantitative reverse transcriptase PCR (qRT-PCR) was performed using 10 μl reaction volume containing 5 μl Syber green (Sso-Advanced Universal SYBR Green Supermix Kit; Bio-Rad), 3 μl distilled H_2_O, 0.5 μl forward primer, 0.5 μl reverse primer, and 1 μl of 1:10 diluted cDNA on the CFX96 Real-Time System (Bio-Rad). Each reaction was subjected to the following conditions: 98 °C for 10 minutes, followed by 44 cycles of 98 °C for 30 seconds, 60 °C for 20 seconds, and 72 °C for 30 seconds in 96-well optical reaction plates (Bio-Rad). The reference gene *GAPDH* was used to normalize the amplification of the target genes. Each qRT-PCR analysis was performed in triplicate. Primer sequences are listed in Additional file 1.

### Western blotting

Cells were treated with 0 and 500 nM JQ1 for 72 hours. Protein was extracted by lysing the cells in RIPA buffer with PMSF (Sigma). Then 10 μl total proteins were separated with SDS-PAGE with 12 % resolving gel and 6 % stacking gel. Samples were diluted in sample diluting buffer, heated at 95 °C for 5 minutes, and loaded on to the gel and electrophoresed. Proteins were then electrophoretically transferred onto Polyvinylidene difluoride (PVDF) membrane for 1 hour at 150 mA constant current. The membrane was blocked with 5 % nonfat dry milk for 1 hour at room temperature and then incubated overnight at 4 °C with primary antibodies for Caspase-8, Caspase-9, and β-actin (Santa Cruz Biotechnology) diluted 1:50 in blocking solution. The membrane was washed to remove unbound primary antibody and was incubated with secondary antibody (1:1000 dilution; Santa Cruz Biotechnology) in blocking solution for 1 hour at 37 °C. Unbound secondary antibody was washed with PBS containing 0.1 % Tween-20. Blots were developed with chemiluminescence system (Bio-Rad), and exposed to X-ray film (FUJI Film, Tokyo, Japan). Band intensity was quantified with ImageJ software, (NIH, Bethesda, MD, USA).

### Statistical analysis

Data are presented as the mean ± standard error of the mean (SEM). Results with *p* ≤0.05 were considered statistically significant. All analyses were performed using SPSS (Chicago, IL, USA) version 11.5 (SPSS Inc., USA).

## Results

### Effect of JQ1 on growth of MSCs

**Fig. 1:**
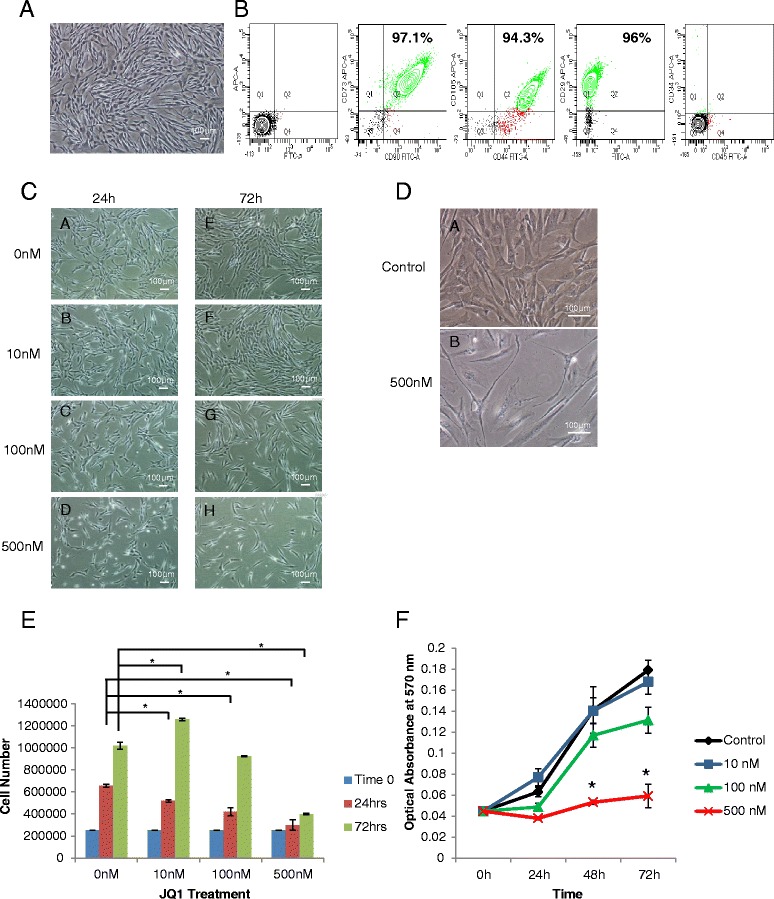
Characterization of cord-derived MSCs and effect of JQ1 on their growth. **a** Phase contrast image of the cord-derived MSCs showing fibroblastoid morphology. Images acquired at 4× magnification. **b** Flow cytometric analysis for cell specific surface markers. Cord-derived MSCs were positive for MSC markers CD90, CD73, CD44, CD105, and CD29 and negative for hematopoietic stem cell markers CD34 and CD45. **c** Effect of JQ1 on cell morphology. Phase contrast images of cells treated with 0, 10, 100, and 500 nM for 24 hours *a*–*d* and for 72 hours *e*–*h*. Images acquired at 4× magnification. **d** Light micrographic images of cells treated with 0 nM *a* and 500 nM *b* JQ1 when cultured for 7 days. Images acquired at 10× magnification. MSCs lost fibroblastoid morphology and grew larger in size upon exposure to JQ1. **e** Effect of JQ1 on growth rate. JQ1 at concentrations of 100 and 500 nM resulted in a significant decrease in cell growth in comparison with the control cells in a time-dependent manner, while 10 nM showed no effect on cellular growth at 24 hours but showed significant increase at 72 hours. **f** Effect of JQ1 on cell metabolic activity as determined using the MTT assay. JQ1 at a concentration of 500 nM resulted in significant decrease in cell growth in comparison with the control cells, while 10 nM showed no effect on cellular growth. *p ≤0.05. *FITC* fluorescein isothiocyanate, *JQ1* (S)-tert-butyl2-(4-(4-chlorophenyl)-2,3,9-trimethyl-6H-thieno[3,2-f][1,2,4]triazolo[4,3-a][1,4]diazepin-6-yl)acetate

MSCs isolated from umbilical cord had fibroblastoid morphology (Fig. 1a) and were positive for the MSC specific surface markers CD90, CD73, CD44, CD105, and CD29, as shown in Fig. 1b. These MSCs exhibited self-renewal and, unlike cancer cells, were capable of differentiation into various lineages. An ideal anticancer drug would inhibit growth or promote apoptosis of cancer cells, but not normal cells, particularly stem cells and progenitors such as MSCs. While JQ1 is well known to cause growth inhibition and apoptosis induction in cancer cells, its effect on normal cells is not well understood. Our studies with JQ1 showed that it affected the morphology of MSCs (Fig. 1c) and caused significant reduction in cell proliferation at the 500 nM dose. When MSCs were treated with 500 nM for 7 days, cells appeared to be significantly larger and flatter than the control cells (Fig. 1d), suggesting cell differentiation. Further analysis of the JQ1-treated cells showed a dose-dependent effect of JQ1 on MSC proliferation (Fig. 1e, f). Evidently, the metabolic activity of MSCs was decreased at 100 and 500 nM JQ1, but not at 10 nM JQ1. In fact, a lower dose of 10 nM JQ1 promoted cell proliferation 24 hours after treatment.

**Fig. 2:**
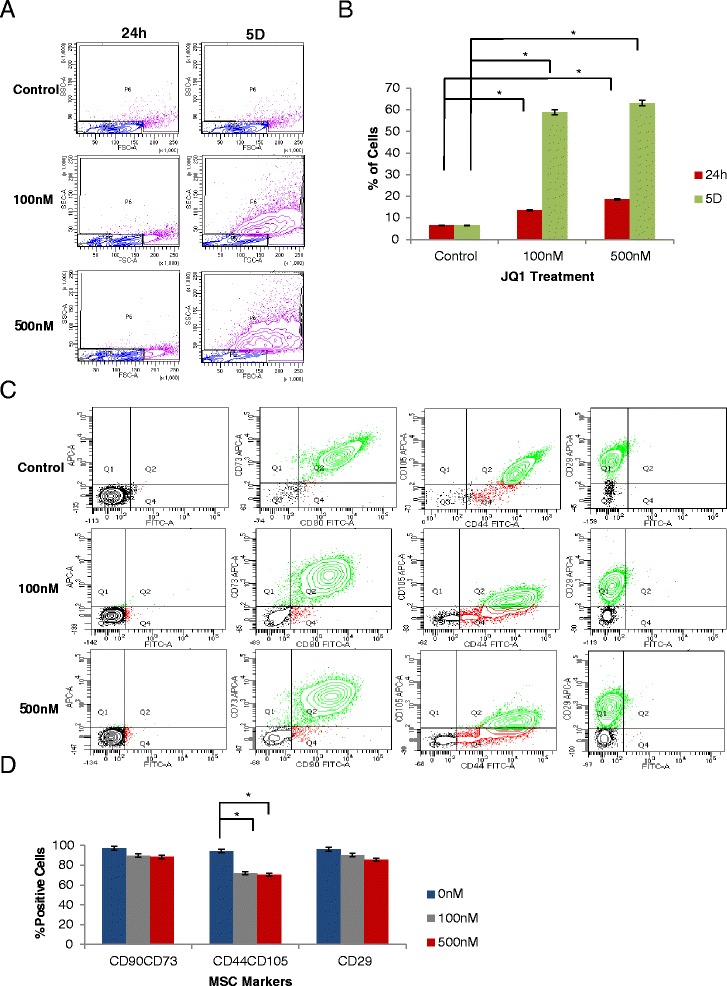
Exposure to JQ1 caused an increase in MSC size with simultaneous loss of CD105. Cells were treated with 0, 100, and 500 nM JQ1 for 24 hours and 5 days and were subjected to FACs analysis. **a** Plots displaying size and granularity of cells. Both size and granularity of cells were increased in a concentration and time-dependent manner. **b** Quantification of percentage of cell populations changed in cell size upon exposure to JQ1. **c** CD105 loss was proportional to concentration of JQ1. **d** Quantification of the MSC surface markers of JQ1 upon 5 days of exposure. *p ≤0.05. All plots are representative of three biological experiments. *JQ1* (S)-tert-butyl2-(4-(4-chlorophenyl)-2,3,9-trimethyl-6H-thieno[3,2-f][1,2,4]triazolo[4,3-a][1,4]diazepin-6-yl)acetate, *MSC* mesenchymal stem cell

Changes in the morphology of the JQ1-treated MSCs were further quantified by flow cytometry. Cell size and granularity increased in a concentration and time-dependent manner (Fig. 2a). Exposure of cells to 100 nM and 500 nM JQ1 for 24 hours resulted in increased cell size in 6 % and 12 % of the populations, respectively, without noticeable effect on the cellular granularity. When the cells were exposed to 100 nM and 500 nM JQ1 for 5 days, both size and granularity were increased in 52 % and 57 % of the cell populations, respectively (Fig. 2b). Although loss of CD90, CD73, CD44, and CD29 was insignificant, CD105 was lost in 22 % and 24 % of MSCs treated with 100 nM and 500 nM JQ1, respectively, for 5 days (Fig. 2c, d).

**Fig. 3:**
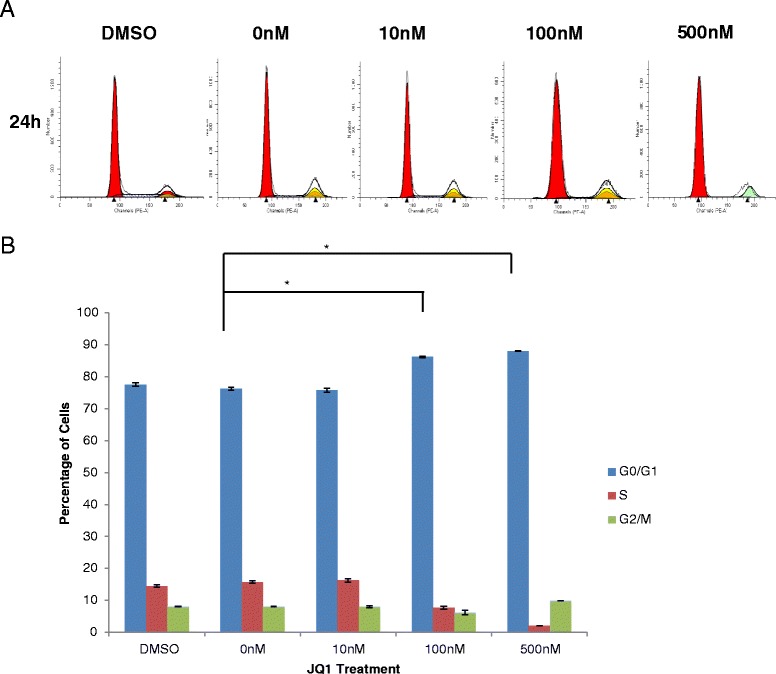
JQ1 arrested MSCs in the G1 phase of the cell cycle. Cell cycle profile of MSCs treated with DMSO and 0, 10, 100, and 500 nM JQ1 for 24 hours, stained with PI. Mean cell population percentage ± SEM (*n* = 3). Analysis was performed using ModFit software (Verity Software House, Topsham, ME, USA). Shown are histogram and graphical representations of cells in the G0/G1, S, and G2/M phases of cell cycle (**a** and **b**, respectively). *p ≤0.05. *DMSO* dimethyl sulfoxide, *JQ1* (S)-tert-butyl2-(4-(4-chlorophenyl)-2,3,9-trimethyl-6H-thieno[3,2-f][1,2,4]triazolo[4,3-a][1,4]diazepin-6-yl)acetate

To test the effect of JQ1 on cell cycle progression, cell cycle analysis was performed on the JQ1-treated MSCs and the results depicted in Fig. 3 show that 100 nM and 500 nM doses of JQ1 had 10 % and 12 % increase in cells arrested in G1 phase, respectively. Conversely, the number of cells in the S phase was decreased in a dose-dependent manner. However, no significant change was observed in the percentage of cells in the G2/M phase in MSCs treated with JQ1. These results suggested that JQ1 induced G1 phase cell cycle arrest in MSCs.

### Analysis of genes involved in cell cycle regulation in JQ1-treated MSCs

**Fig. 4:**
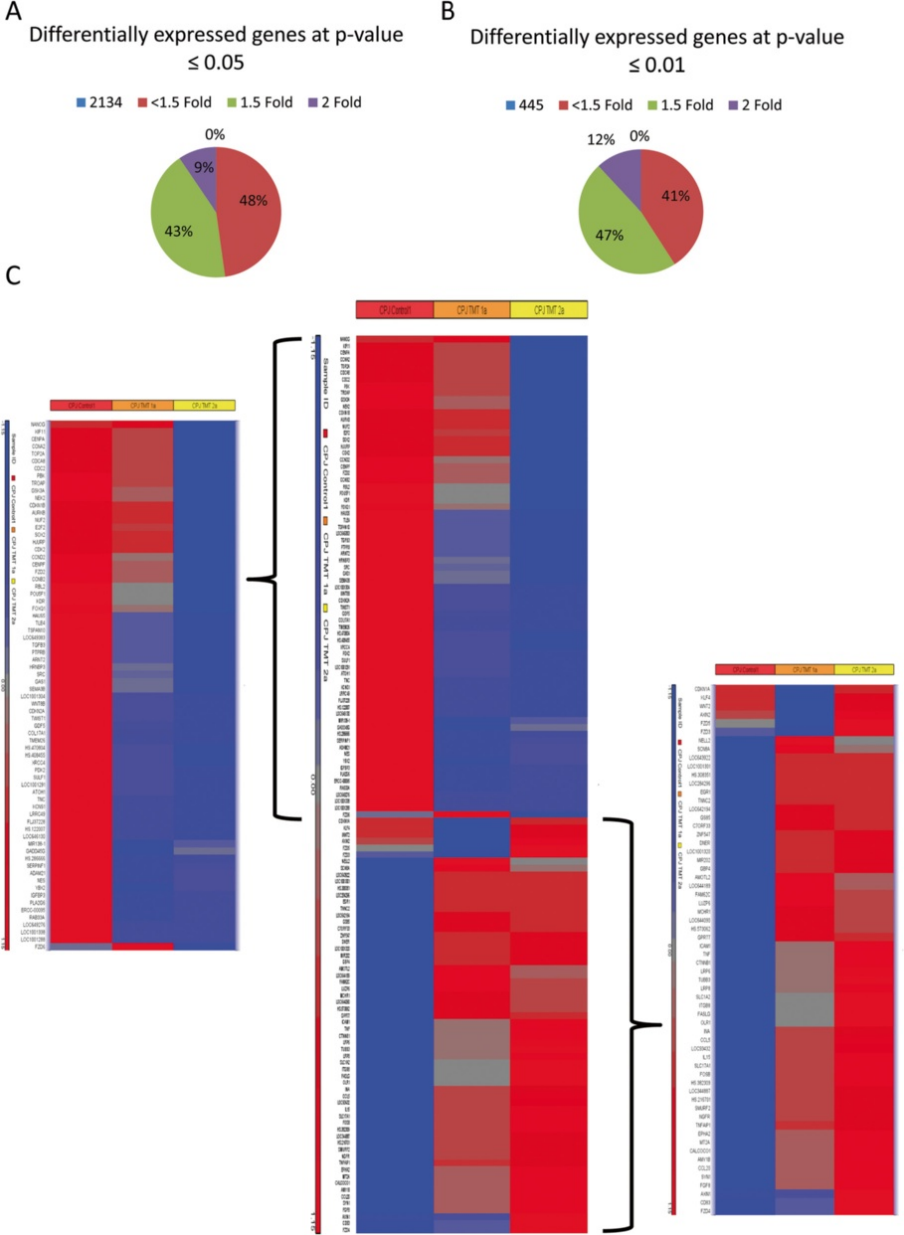
Microarray analysis of MSCs treated with JQ1. Pie charts showing the percentage of differentially expressed genes in JQ1-treated MSCs at different fold cutoff values at *p* ≤0.05 **a** and *p* ≤0.01**b. c** Heatmap depicting highly upregulated (*red*) and downregulated (*blue*) genes, in MSCs treated with 0, 100, and 500 nM JQ1 for 24 hours. Zoomed-in downregulated genes (*left*) and upregulated genes (*right*)

To investigate the global effect of JQ1 on gene expression in MSCs, microarray analysis was performed on cells treated with 0, 100 and 500 nM JQ1 for 24 hours. Results showed that 2134 genes were differentially upregulated or downregulated; 445 of them were differentially expressed with *p* ≥0.01 (210 genes were expressed within 1.5-fold cutoff values and 53 at 2-fold cutoff values) (Fig. 4a, b). Further analysis showed that one of the highly regulated groups of genes was those which are involved in cell cycle regulation, including *CCNA2*, *CCNB2*, *CDC2*, and *E2F2* genes (Fig. 4c). In addition, several genes involved in DNA replication and regulation of mitosis and cell division, such as *TOP2A*, *CDCA8*, *NEK2*, *CENPF*, *CENPA*, *PBK*, and *AURKB*, as well as genes involved in senescence such as *RB2*, *CDKN1A*, *CDKN2A*, and *CDKN1B*, were significantly downregulated.

**Fig. 5:**
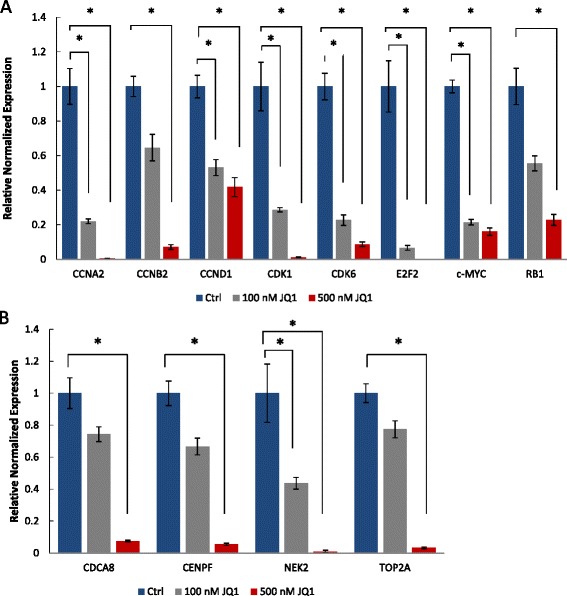
Downregulation of genes in MSCs treated with 100 and 500 nM JQ1 for 24 hours. **a** qRT-PCR analysis of transcripts of cell cycle genes including *CCNA2*, *CCNB2*, *CCND1*, *CDK1*, *CDK6*, *E2F2*, *c-MYC*, and *RB1*. **b** DNA replication and mitosis genes including *CDCA8*, *CENPF*, *NEK2*, and *TOP2A*. *p ≤0.05. *Ctrl* control, *JQ1* (S)-tert-butyl2-(4-(4-chlorophenyl)-2,3,9-trimethyl-6H-thieno[3,2-f][1,2,4]triazolo[4,3-a][1,4]diazepin-6-yl)acetate

Downregulation of these genes in MSCs treated with 100 and 500 nM JQ1 for 24 hours was further investigated by qRT-PCR. Results depicted in Fig. 5 showed that 100 nM JQ1 caused a 3-fold to 9-fold decrease in *CCNA2*, *CDK1*, *E2F2*, and *CCNB2* transcription in comparison with the control, and 500 nM JQ1 led to more than a 9-fold decrease in the transcription of these genes (Fig. 5a). Furthermore, the transcriptional levels of *CCND1*, *CDK6*, *c-MYC*, and *RB1* were found to be downregulated 4-fold to 9-fold compared with control when examined by qRT-PCR. Among these genes, *c-MYC* transcription was downregulated 8-fold, while the transcription of *CCND1* decreased 5-fold to 6-fold in JQ1-treated cells. *CDK6* transcription, however, was downregulated by 8-fold to 9-fold. *RB1* transcription also decreased in a dose-dependent manner from 4-fold to 8-fold as shown in Fig. 4a. Moreover, 500 nM JQ1 caused significant reduction (approximately 9-fold) in the transcription of *TOP2A*, *CDCA8*, *NEK2*, and *CENPF* (Fig. 5b). Taken together, these results showed downregulation of genes involved in cell cycle regulation.

### Effect of JQ1 on cell apoptosis

**Fig. 6:**
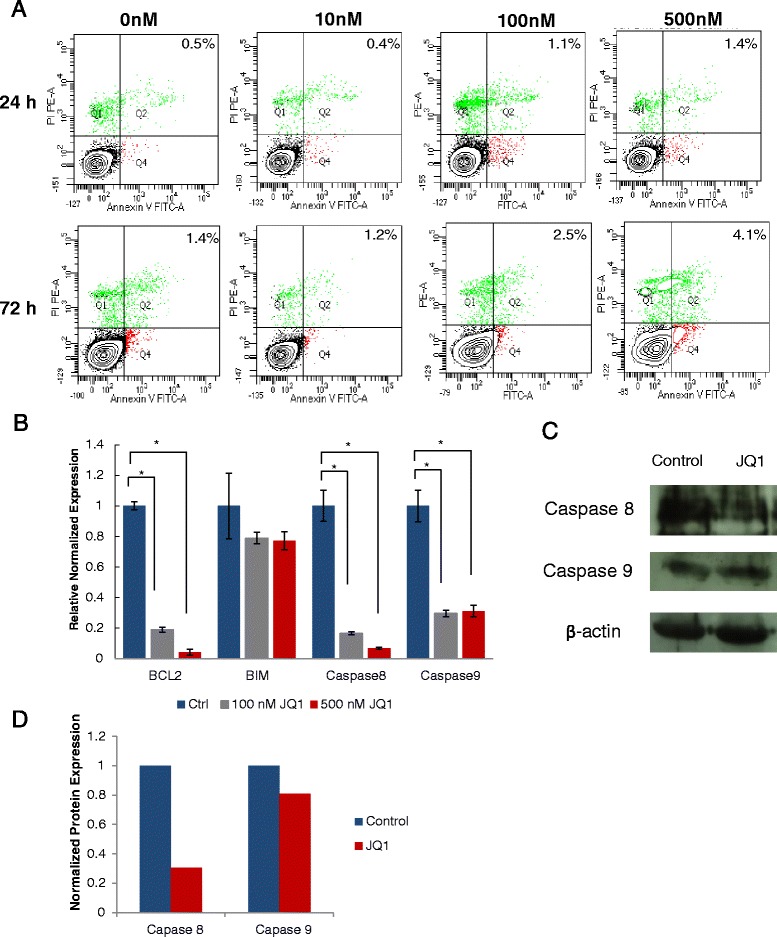
Effect of JQ1 on apoptosis of MSCs. **a** Annexin V/FITC and PI staining of MSCs treated with 0, 10, 100 and 500 nM JQ1 for 24 and 72 hours. Plots are representative of the mean number of Annexin V-positive cells (*n* = 3) from three biological experiments. **b** qRT-PCR analysis of apoptosis genes including *BCL2*, *BIM*, *Caspase 8*, and *Caspase 9* **c**. *p ≤0.05. **c** Western blotting showing downregulation of Caspase 8 and Caspase 9 in MSCs treated with 500 nM JQ1. **d** Caspase 8 and 9 expression was quantified by ImageJ software and normalized to β-actin expression. *Ctrl* control, *FITC* fluorescein isothiocyanate, *JQ1* (S)-tert-butyl2-(4-(4-chlorophenyl)-2,3,9-trimethyl-6H-thieno[3,2-f][1,2,4]triazolo[4,3-a][1,4]diazepin-6-yl)acetate, *PI* propidium iodide

MSCs treated with JQ1 had no observable cell death. To further investigate whether JQ1 induced apoptosis in MSCs, Annexin V-treated cells were analyzed by flow cytometry. These results, as depicted in Fig. 6a, showed that JQ1 treatment for up to 72 hours had no significant increase in apoptotic cell population. These cells were also analyzed for expression of genes involved in regulation of apoptosis including antiapoptotic *BCL2*, proapoptotic *BIM*, and initiator Caspases 8 and 9. Results showed that mRNA expression levels of *BCL2*, Caspase 8 and Caspase 9 were reduced significantly by 6-fold to 9-fold while no significant change in the mRNA expression of *BIM* was observed (Fig. 6b). Further investigation by western blot analysis indicated that levels of proapoptotic proteins such as Caspase 8 and Caspase 9 were reduced in JQ1-treated cells in comparison with the control (Fig. 6c, d). However, the reduction in expression of Caspase 9 was less pronounced (Fig. 6d). These results suggested that MSCs did not undergo apoptosis in response to JQ1.

### Effect of JQ1 on expression of genes involved in WNT signaling and differentiation

**Fig. 7:**
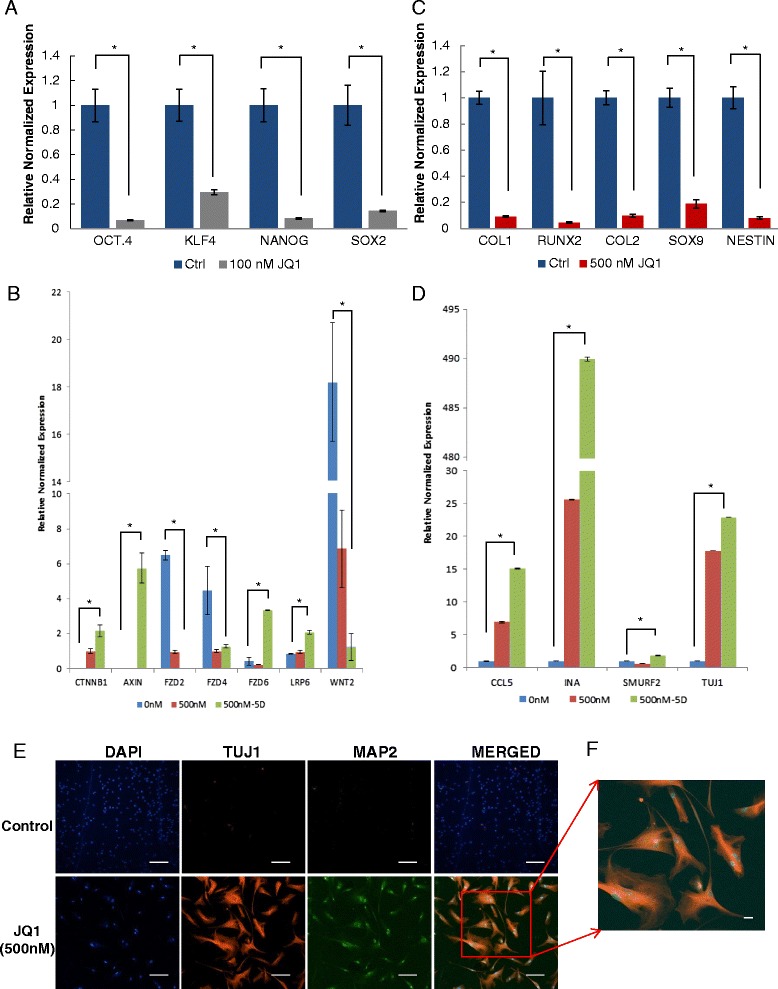
Effect of JQ1 on the expression of pluripotency, WNT signaling, and differentiation genes as determined by qRT-PCR **a**–**d** and immunocytochemical analysis **e**. **a** Pluripotent markers *OCT4*, *KLF4*, *Nanog*, and *SOX2* were downregulated in MSCs treated with 100 nM JQ1 for 24 hours. **b** WNT signaling genes *CTNNB1*, *AXIN*, *FZD6*, and *LRP6* were upregulated and *WNT2*, *FZD2* and *FZD4* were downregulated in MSCs treated with 500 nM JQ1 for 24 hours and 5 days. **c** Osteogenic markers *COL1* and *RUNX2*, chondrogenic markers *COL2* and *SOX9*, and neurogenic marker *Nestin* were downregulated in MSCs treated with 500 nM JQ1 for 24 hours. **d** Neuronal markers *TUJ1*, *CCL5*, *INA* and *SMURF2* were upregulated in MSCs treated with 500 nM JQ1 for 24 hours and 5 days. **e** TUJ1 and MAP2 proteins were expressed in MSCs treated with 500 nM JQ1 for 5 days. Images acquired at 10× magnification. **f** Zoomed-in merged image as shown in **e**. *p ≤0.05. *Ctrl* control, *DAPI* 4′,6-diamidino-2-phenylindole, *JQ1* (S)-tert-butyl2-(4-(4-chlorophenyl)-2,3,9-trimethyl-6H-thieno[3,2-f][1,2,4]triazolo[4,3-a][1,4]diazepin-6-yl)acetate

To test whether JQ1 induces the differentiation of MSCs, microarray data indicated downregulation of genes involved in self-renewal (Fig. 4c). Significant downregulation of *Oct4*, *Nanog*, *Sox2*, and *Klf4* markers in MSCs treated with JQ1 was confirmed by qRT-PCR (Fig. 7a). This led us to look at the WNT signaling pathway, which is involved in regulating the genes involved in self-renewal and differentiation. Downregulation of WNT genes is known to indicate decreased self-renewal and increased differentiation. Determination of the transcriptional level of WNT genes showed that *WNT4*, *WNT5a*, and *WNT11* were not expressed in MSCs and were not affected by JQ1. However, the expression of *WNT2*, a member of the canonical WNT pathway, was decreased by 8.5-fold and 9.5-fold in MSCs treated with 100 nM and 500 nM JQ1, respectively, compared with control. This is in agreement with the microarray analysis, which showed that the expression of *FZD2*, a WNT receptor involved in the canonical WNT pathway, and *WNT2* were also downregulated in JQ1-treated cells (Fig. 4c). Further analysis revealed downregulation of *FZD4* but a 2-fold upregulation of *FZD6* and *LRP6* compared with the control. In addition, WNT-related proteins CTNNB1 and AXIN were upregulated by 2-fold and 6-fold, respectively. Taken together, these results suggest JQ1 modulated signal transduction by turning off WNT pathway resulting in downregulation of pluripotent genes and induction of differentiation (Fig. 7b). These results prompted us to explore the transcription levels of lineage-specific differentiation markers. Results depicted in Fig. 7c showed that osteogenic markers *Col1* and *Runx2*, and chrondrogenic markers *Col2* and *Sox9*, were downregulated. Interestingly, *Nestin* was downregulated but *TUJ1*, *CCL5*, *INA*, and *SMURF2* were significantly upregulated (Fig. 7d). Microarray data were also in agreement with qRT-PCR results showing upregulation of all these genes. Furthermore, immunocytochemical analysis confirmed the expression of these specific proteins, TUJ1 and MAP2 (Fig. 7e). While expressions of these genes are generally associated with neuronal differentiation, additional studies will be required to show a direct effect of JQ1 on differentiation of MSCs.

## Discussion

A number of chemicals have been developed and investigated for their anticancer properties, such as cell cycle arrest or cell death of cancer cells [4, 6]. Potential anticancer agents are typically tested in cultured cancer cells and animal models [19]. Studying the safety of anticancer agents in animal models has limitations because the results may not be directly applicable to humans due to genetic and physiological differences. For instance, only one-third of drugs tested using animal models pass onto clinical trials [20]. For these reasons, it is important to develop alternative methods, which have greater predictability and are less expensive to test safety and efficacy of new drugs. In this study, we use MSCs isolated from human umbilical cord tissue as a model system to test the safety of a potential anticancer agent, JQ1, which has been extensively investigated in animal models and various cancer cell lines [11, 12, 14]. It has been shown that JQ1, used in concentrations ranging from 100 nM to 1 μM, exerted anticancer activities in cancer cell lines through the inhibition of BET proteins, and consequently alteration in the transcription of target genes which play pivotal roles in the progression of cancerous diseases [21–23]. However, its effect on normal stem cells such as MSCs, which are involved in repair and regeneration of damaged tissues, is poorly studied both in vivo and in vitro.

Our study showed that JQ1 affected cell morphology and inhibited proliferation of cord-derived MSCs. This observation was consistent with the growth inhibition caused by JQ1 in various cancer cell lines derived from NMC, multiple myeloma, leukemia, and neuroblastoma [11–15]. JQ1 caused increases in cell size and granularity with simultaneous loss of CD105, suggesting differentiation of MSCs. Loss of CD105 has been implicated in the differentiation of cord blood-derived MSCs [24]. To explore the mechanism of growth inhibition of MSCs, we performed cell cycle analysis on MSCs treated with JQ1. Cell cycle profiles of JQ1-treated cells showed an increase in the percentage of cells arrested in the G1 phase, but a decrease in the percentage of cells in the S phase. These results indicated that JQ1 adversely affected cell cycle progression in MSCs. These observations are in agreement with previous studies in which JQ1 has been reported to induce G1 cell cycle arrest in many cancer cells, including nerve sheath tumors and leukemic cell lines [6, 17].

Previous studies with cancer cells have shown that JQ1 binds to BET proteins and inhibits their interaction with acetylated histones, causing cell cycle arrest in the G1 phase and apoptosis [11, 12]. Furthermore, JQ1 also downregulates key genes involved in control of the cell cycle and upregulates genes involved in apoptosis [14, 15, 17, 25]. Therefore, we questioned whether genes involved in cell cycle regulation and apoptosis were affected by JQ1 in MSCs as well.

Microarray analysis of JQ1-treated MSCs pointed to cell cycle arrest in the G1 phase. Microarray data confirmed by qRT-PCR analyses showed that a number of genes involved in cell cycle regulation, including *CCNA2*, *CCNB2*, *CCND1*, *CDK1*, *CDK6*, *c-MYC*, and *E2F2*, were downregulated. *CCNA2* interacts with *CDK2*, and their activity starts late in the G1 phase and lasts through the S and G2 phases [26, 27]. BRD2 has been shown to activate the transcription of *CCNA2* through binding to its promoter [28, 29]. Downregulation of *CCNA2* in our study is consistent with the observation that overexpression of BRD2 upregulated the transcription of *CCNA2* which led to aggressive cancer in the mouse [30]. Also, a BRD2 mutation resulted in loss of *CCNA2* transcriptional activation in NIH3T3 cells [31, 32]. *CCND1* and *CCND2* are known to play a role in G1/S transition, and they associate with their partners *CDK4* and *CDK6* during cell cycle regulation [33, 34]. Cyclin D is required to drive the cell cycle through the transcription activation of Cyclin A and Cyclin E. As such, they are connected to many cancers through their overexpression or the interruption of their pathways [35]. Studies showed that JQ1 or BRD4 knockdown in NF1 (Neurofibromatosis type 1)-associated malignant peripheral nerve sheath tumors decreased the expression of *CCND1* [16]. A similar effect was observed upon treatment of medulloblastoma cell lines with JQ1 [36]. The effect of JQ1 on MSCs in our study, as well as on cancer cell lines in the other studies, are in line with the previous studies showing that BRD4 activates the transcription of *CCND1* and *CCND2*, and that their expression is reduced in NIH3T3 cells after knockdown of BRD4 [33]. BRD2 also has been shown to regulate the transcription of *CCND1* [28, 29]. *c-MYC* is an oncogene that is expressed early in the G1 stage and has a crucial role in cell cycle progression [37]. It is known to activate the transcription of proliferation genes, and overexpression is associated with tumorigenesis [36, 38–40]. *c-MYC* has been shown to be regulated by BRD4 in various cancer cell lines such as Hela cells and leukemia cells, and *c-MYC* expression was reduced upon BRD4 knockdown or cell treatment with small molecules inhibiting BRD4 [14, 37]. JQ1 is known to interfere with BRD4, consequently decreasing c-MYC expression in MSCs, which was evident in our results. The *E2F* transcription factor family was among the highly downregulated groups in the gene expression profile. We confirmed that *E2F2* transcription reduced upon JQ1 treatment of MSCs with qRT-PCR. The E2F family plays a critical role in the regulation of the cell cycle through transcriptional regulation of genes involved in the G1 phase and DNA replication [41]. BRD2 has been shown to form a complex with *E2F* and transactivates E2F responsive genes in NIH3T3 cells [28, 29, 32, 41]. *CCND1*, *CCNA*, *CCNE*, and dihydrofolate reductase were among the *E2F* responsive genes [28, 29, 32]. Although G1 arrest, along with downregulation of *RB1* and stemness genes, has been reported in senescent cells [42], our results showed downregulation of senescence genes such as *RB2*, *CDKN1A*, *CDKN2A*, and *CDKN1B*, suggesting that JQ1-induced arrest in MSCs is not due to senescence.

Since JQ1 resulted in cell cycle arrest in MSCs, we further investigated whether JQ1 induced apoptosis as reported in cancer cells. Flow cytometric analysis also showed no significant increase in Annexin V-positive cells, suggesting that JQ1 did not induce apoptosis in MSCs. To further confirm that JQ1 did not exert an apoptotic effect in MSCs, transcriptional levels of *BCL2*, *BIM*, *Caspase 8*, and *Caspase 9*, which play critical roles in controlling the apoptotic pathways, were determined by qRT-PCR. Even though JQ1 caused a significant downregulation of *BCL2*, an antiapoptotic marker, expression of proapoptotic genes *Caspase 8* and *Caspase 9* were also downregulated, while no significant change in the expression of *BIM* was detected. Western blot analysis also revealed significant downregulation of Caspase 8 but little change in the expression of Caspase 9, which is consistent with the qRT-PCR analysis as discussed. These results suggested that MSCs did not undergo apoptosis when they were subjected to JQ1 treatment.

Stem cells have been reported to express several self-renewal markers, including *OCT4*, *Nanog*, *KLF4*, and *SOX2* [43–47]. Our microarray and qRT-PCR data showed that JQ1 treatment of MSCs led to downregulation of *OCT4*, *Nanog*, *KLF4*, and *SOX2*, suggesting that BET proteins play an essential role in the self-renewal of MSCs.

Since signaling pathways are known to play an important role in differentiation processes, we investigated whether WNT signaling was involved in self-renewal and differentiation of stem cells [48]. Microarray analysis of the transcripts from JQ1-treated MSCs indicated differential expression of WNT-related genes. Further analysis of transcripts of JQ1-treated cells by qRT-PCR revealed downregulation of *WNT2*, *FZD2,* and *FZD4* and upregulation of *FZD6*, *LRP6*, *CTNNB1*, and *AXIN*. These observations suggest that the WNT pathway is turned off and differentiation of MSCs is induced by JQ1. This is in partial agreement with a previous report where downregulation of *WNT2*, *FZD2*, and *FZD4* and upregulation of *CTNNB1* was involved in neural differentiation of human adipose-derived stem cells [49]. Taken together, these observations suggested that JQ1 modulates WNT signaling, which is known to be involved in self-renewal and differentiation of MSCs.

It has been proposed that BET proteins exert a dual function where they act as coactivators for cell cycle genes and corepressors for specific differentiation markers [1]. Therefore, we investigated the transcriptional expression of lineage-specific differentiation markers. Among all of the investigated differentiation markers, only *TUJ1*, *CCL5*, *INA*, and *SMURF2* were upregulated, which are neuronal lineage-specific markers [50–52]. Although these data showed modulation of suggested neuronal markers, this alone is not sufficient to implicate JQ1 in neural/neuronal differentiation. Nevertheless, the results indicate the harmful effects of JQ1 on MSCs by modulating not only the WNT signaling but also the genes involved in the differentiation. Moreover, significant upregulation of genes such as *TNF*, *CCL5*, and *ICAM1* is interesting as observed by microarray analysis because these genes have been reported to be important for leukocyte recruitment and cell adhesion [53, 54], suggesting that JQ1 treatment of MSCs may have implication in tumor progression in situ.

Our results clearly showed that JQ1 inhibited MSC growth by influencing important cellular processes including cell cycle, signal transduction, and differentiation. In addition, genes involved in self-renewal, DNA replication, and mitosis were downregulated. JQ1 is likely to exert a similar effect on MSCs derived from other adult sources such as bone marrow MSCs. Interestingly, recently published reports showed antioxidant activity of JQ1 and its ability to block memory in mice [55, 56]. Therefore, further studies are needed to clarify the molecular mechanism of action of the BET proteins in MSCs. Since JQ1 can inhibit all BET proteins, and a redundancy in the BET proteins function has been proposed, additional investigation would help in elucidating which one of the BET proteins is responsible for the transcription of genes involved in MSC cell cycle regulation.

## Conclusion

Overall, this study revealed harmful effects of JQ1 and possibly other members of the thienodiazepine class on growth, self-renewal, and cell cycle progression of MSCs. The use of this class of BET inhibitors as anticancer agents should therefore be more carefully evaluated before their use for cancer therapy. Furthermore, our studies provided a promising model to supplement animal studies to investigate the safety of anticancer agents or drugs. This is a simple and inexpensive system compared with animal models and could provide results and predictions directly applicable to humans. These findings are likely to have implications in stem cell biology, cancer therapy, and regenerative medicine and provide a basis for future studies.

## Additional file

Additional file 1: Table S1. Presenting a list of human primer sequences used in qRT-PCR. (DOCX 16 kb)

## Abbreviations

BD: Bromodomain
BET: Bromo- and extra-terminal domain
DAPI: 4′,6-Diamidino-2-phenylindole
DMEM: Dulbecco’s modified Eagle’s medium
DMSO: Dimethyl sulfoxide
DNMT3A: DNA methyltransferase 3A
ET: Extra-terminal
FITC: Fluorescein isothiocyanate
GM: Growth medium
JQ1: (S)-Tert-butyl2-(4-(4-chlorophenyl)-2,3,9-trimethyl-6H-thieno[3,2-f][1,2,4]triazolo[4,3-a][1,4]diazepin-6-yl)acetate
MSC: Mesenchymal stem cell
NMC: NUT midline carcinoma
NUT: Nuclear protein in testis
PBS: Phosphate-buffered saline
PI: Propidium iodide
qRT-PCR: Quantitative reverse transcriptase PCR
SEM: Standard error of the mean
STAT5: Signal transducer and activator of transcription 5
