# Supplementary material for: BET protein inhibitor JQ1 inhibits growth and modulates WNT signaling in mesenchymal stem cells
Source: Stem Cell Res Ther. 2016 Feb 1;7:22. doi: 10.1186/s13287-016-0278-3 (PMC4736146; doi:10.1186/s13287-016-0278-3)
Supplement: Additional file 1: Table S1. — Presenting a list of human primer sequences used in qRT-PCR. (DOCX 16 kb) [file 13287_2016_278_MOESM1_ESM.docx]

**Additional file 1: Table S1**

*List of Human Primer Sequences Used in qRT-PCR.*

| Gene | Primer Sequence | | |
| --- | --- | --- | --- |
|  | Forward (5’-3’) | Reverse (5’-3’) | Product Length |
| *BCL2* | GGATGCCTTTGTGGAACTGTA | CCAAACTGAGCAGAGTCTTCA | 86 |
| *BIM* | AGTGGGTATTTCTCTTTTGACACAG | GTCTCCAATACGCCGCAACT | 192 |
| *Caspase 8* | CCAGAGACTCCAGGAAAAGAGA | GATAGAGCATGACCCTGTAGGC | 185 |
| *Caspase 9* | AACAGGCAAGCAGCAAAGTT | CACGGCAGAAGTTCACATTG | 247 |
| *CCNA2* | GGTACTGAAGTCCGGGAACC | GTGACATGCTCATCATTTACAGG | 105 |
| *CCNB2* | CCGACGGTGTCCAGTGATTT | TGTTGTTTTGGTGGGTTGAACT | 180 |
| *CCND1* | CTGTGCATCTACACCGACAACTC | AGGTTCCACTTGAGCTTGTTCAC | 86 |
| *CDCA8* | CTTCGCCCTTGGAGGAAACAA | GGTGTCTGAATAGCTTCTGCTG | 102 |
| *CDK1* | CATGGCTACCACTTGACCTGT | AAGCCGGGATCTACCATACC | 128 |
| *CDK6* | AGACCCAAGAAGCAGTGTGG | AAGGAGCAAGAGCATTCAGC | 387 |
| *CENPF* | AGAACCCACCACGAAATCC | TGCCTTCACTGGACCTTACA | 162 |
| *c-MYC* | CGGAACTCTTGTGCGTAAGG | CTCAGCCAAGGTTGTGAGGT | 123 |
| *COL1* | AAGGTCATGCTGGTCTTGCT | GACCCTGTTCACCTTTTCCA | 114 |
| *COL2* | CTCGTGGCAGAGATGGAGAA | CACCAGGTTCACCAGGATTG | 252 |
| *E2F2* | GGCCAAGAACAACATCCAGT | TGTCCTCAGTCAGGTGCTTG | 170 |
| *GAPDH* | ACAACTTTGGTATCGTGGAAGG | GCCATCACGCCACAGTTTC | 101 |
| *KLF4* | CGAACCCACACAGGTGAGAA | TACGGTAGTGCCTGGTCAGTTC | 94 |
| *Nanog* | AAAGAATCTTCACCTATGCC | GAAGGAAGAGGAGAGACAGT | 110 |
| *NEFL* | GGATCGTTGATGCCCAGTCT | CGGATGGACTTGAGGTCGTT | 77 |
| *NEK2* | AAGGAATGCCACAGACGAAG | TGCGATTCATTTGTTCAGGA | 199 |
| *Nestin* | CAACTTGGAGGGGAAGTCAC | CTCAGAGACTAGCGGCATTC | 97 |
| *OCT4* | CCCCTGGTGCCGTGAA | GCAAATTGCTCGAGTTCTTTCTG | 97 |
| *FZD2* | TTTCTGGGCGAGCGTGAT | AAACGCGTCTCCTCCTGTGA | 86 |
| *FZD4* | GGCGGCATGTGTCTTTCAGT | GAATTTGCTGCAGTTCAGACTCTCT | 76 |
| *FZD6* | ACTCTTGCCACTGTGCCTTT | GCACAAGATACAAGCCGCTG | 167 |
| *LRP6* | GCCAGAGGATGTTGTGTCCA | AAGAGGCACAGAAGCTGGTC | 89 |
| *CTNNB1* | GAAACGGCTTTCAGTTGAGC | CTGGCCATATCCACCAGAGT | 166 |
| *AXIN1* | TGGACCTCGGAGCAAGTTTC | CTGTGGACACCAGTTCTCCC | 78 |
| *CCL5* | TACCATGAAGGTCTCCGC | GACAAAGACGACTGCTGG | 199 |
| *INA* | ACCAAGAGTGAGATGGCACG | TTGGGAAGTGGATTCAGCCC | 164 |
| *SMURF2* | CCGAATAGGCACAGGAGGAC | GCGGTTCTCCTTTCTTCCCA | 87 |
